# Room-temperature spinor condensate in halide perovskite microcavity

**DOI:** 10.1126/sciadv.aeb1521

**Published:** 2026-05-22

**Authors:** Takaya Inukai, Ryohei Shibano, Taiki Ogura, Tohki Inoue, Daichi Okada, Shun Takahashi, Kenichi Yamashita

**Affiliations:** Faculty of Electrical Engineering and Electronics, Kyoto Institute of Technology, Matsugasaki, Sakyo-ku, Kyoto 606-8585, Japan.

## Abstract

A deeper understanding of room temperature polariton condensed phases is essential for advancing quantum applications. Spin degrees of freedom inherent in polariton particles manifest themselves in the form of spinor condensation, which has been demonstrated only at cryogenic temperatures in the past. Herein, we demonstrate room-temperature spinor polariton condensation in a lead-halide perovskite microcavity under nonresonant optical excitation. The crystalline anisotropy of the perovskite induces a linear polarization splitting of the lower polariton modes, and, above the condensation threshold, nonlinear polariton-polariton interactions drive the formation of elliptically polarized condensates. The condensation dynamics are analyzed using a spin-dependent Gross-Pitaevskii model, which provides a qualitative framework for understanding the experimentally observed polarization evolution and two-stage threshold behavior. Our findings pave the way for all-optical control of polariton spin states at room temperature, opening a path toward scalable polaritonic quantum devices.

## Teaser

Low-threshold spinor condensate is achieved at room temperature by strong polariton-polariton interactions in CsPbBr_3_ microcavity.

## INTRODUCTION

Microcavity polaritons are quasiparticles that arise from the strong coupling between light and matter. Their hybrid nature opens up a variety of new possibilities in the fields of optoelectronics and quantum electronics (*1*, *2*). The coexistence of a small effective mass, originating from their photonic part, and nonlinearity, stemming from their excitonic part, enables Bose-Einstein condensation under relatively moderate conditions, known as polariton condensation (*3*, *4*). The polariton condensed phase, characterized by high spatial coherence, is exploited as a mechanism for long-range coherent energy transport (*5*, *6*). Numerous studies have demonstrated polariton condensation even at room temperature (RT) in various materials (*7*–*10*), highlighting its potential for highly accessible quantum technologies (*11*, *12*).

Polariton particles also inherit spin properties from excitons. The $J_{z}=\pm 1$ spin projections of total angular momentum along the normal direction of the cavity plane are associated with right- and left-circularly polarized light emissions, respectively. This spin degree of freedom of polariton particles is preserved into their condensed phase, leading to spinor condensation (*13*–*21*). In isotropic media, the polariton condensed mode formed under nonresonant pumping consists of equal densities of spin-parallel and spin-antiparallel polariton particles. The superposition of these two spin-polarized components results in linear polarization along a specific in-plane direction. In optically anisotropic media, on the other hand, the degeneracy of linearly polarized modes with orthogonal polarization directions is lifted, leading to symmetry breaking in spin polarization. More specifically, the anisotropy between spin-parallel and spin-antiparallel polariton interactions, combined with the difference in dissipation rates of the degeneracy-lifted linearly polarized condensed modes, induces a phase transition into the circularly polarized polariton condensed regime. As a result, the microcavity system emits circularly polarized light.

Manipulation of polarization state on the Poincaré sphere is a notable challenge for quantum applications of spinor polaritons (*22*–*24*). However, most studies on circularly polarized emission from spinor condensation have been conducted in microcavities operating at cryogenic temperatures (*25*, *26*), with very few demonstrated at RT. A recent study has reported an RT polariton spin switch in a transition metal dichalcogenide microcavity, where spin-anisotropic polariton-polariton interactions were demonstrated under resonant pumping (*21*). However, explicit spinor condensation has not yet been observed for RT polaritons. Confirming this phenomenon in a RT polariton system is of great academic importance.

In this study, we will explicitly show spinor condensation of RT polariton in a lead halide perovskite microcavity under nonresonant optical pumping. Optical anisotropy of CsPbBr_3_ crystal induces splitting of polariton mode into two orthogonal linear polarizations. Increase in pumping fluence results in a nonlinear increase in polariton population. Full polarization tomography measurements reveal that emission from polariton condensed modes includes circularly polarized components, indicating RT spinor condensation. Numerical analysis suggests that substantial polariton-polariton interaction in CsPbBr_3_ microcavity gives rise to spinor condensation at a threshold as low as ~1.2 times that of the initial threshold for conventional polariton condensation. These findings underscore the remarkable quantum properties of RT polariton systems based on lead halide perovskite materials, paving the way for their potential application in next-generation quantum technologies.

## RESULTS

We use a modified solution-based cast-capping method or a modified vapor-phase chemical deposition method to grow high-quality CsPbBr_3_ crystal plates. The CsPbBr_3_ crystals grown by these methods show excitonic absorption peaks at ~2.4 eV with a linewidth of ~50 meV (see fig. S1). To fabricate a microcavity, a platelet single crystal with a thickness of ~1 to 2 μm and a lateral size of ~50 μm by 50 μm is sandwiched between a pair of distributed Bragg reflectors (DBRs; see fig. S2). Figure S3 exhibits microscopic images of CsPbBr_3_ crystals enclosed within the cavity mirrors. The detailed fabrication procedure is described in Materials and Methods and in our recent publications (*27*, *28*). Photoluminescence (PL) dispersion measurements are performed using a Fourier imaging setup. An example of below-threshold result is shown in fig. S4, demonstrating formation of lower polariton (LP) mode. An analysis based on a coupled oscillator model reveals Rabi-splitting energy of ~110 meV, which is comparable to recent results (*27*, *29*–*31*).

**Fig. 1.**
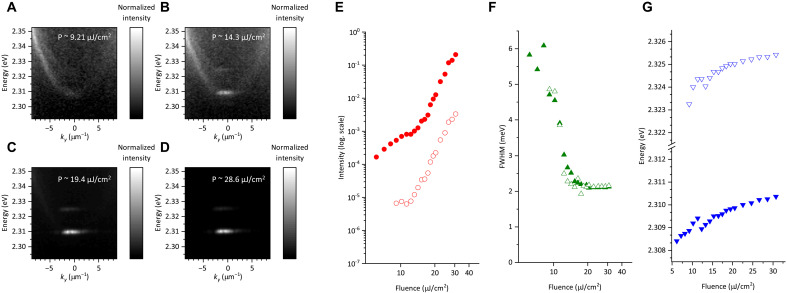
Unpolarized PL results of CsPbBr_3_ microcavity under nonresonant pumping. (**A** to **D**) Grayscale Fourier-imaging maps of PL at pumping fluence P of ~9.21 (A), ~14.3 (B), ~19.4 (C), and ~28.8 μJ/cm^2^ (D). (**E** to **G**) Pumping fluence dependencies of output PL intensity (E), full width at half maximum (FWHM) of PL line at k ~ 0 (F), and PL peak energy (G). Closed and open symbols show data for the first and second LP modes, respectively.

Figure 1 (A to D) shows Fourier-imaging maps of unpolarized PL spectra for a CsPbBr_3_ microcavity under nonresonant optical pumping. The *Q*-factor of this cavity is as small as ~300, facilitating observation of below-threshold PL feature, but the threshold for condensation is relatively high (~14 μJ/cm^2^). At a pump fluence of ~9.21 μJ/cm^2^, we observe a pair of dispersion curves with local minima at ~2.308 and ~2.322 eV (see Fig. 1A). This result indicates a splitting of the LP mode, attributed to the in-plane crystallographic anisotropy of the CsPbBr_3_ crystal (*28*, *32*–*34*). Hereafter, the LP mode with the lower energy is referred to as the first LP mode and the one with the higher energy as the second LP mode. At a higher pump fluence of ~14.3 μJ/cm^2^ (see Fig. 1B), the first LP mode exhibits directional emission, indicating momentum-space condensation of polariton particles. With further increase in pump fluence, condensation also occurs in the second LP mode (~19.4 μJ/cm^2^, see Fig. 1C). At an even higher fluence (~28.6 μJ/cm^2^, see Fig. 1D), blueshifts of the condensed modes are observed, revealing the polariton-polariton nonlinear interactions.

Figure 1 (E to G) presents the pump fluence–dependent behavior of PL from the LP modes. The PL intensities exhibit S-shaped nonlinear responses respect to the fluence (see Fig. 1E). The full width at half maximum of the PL shows pronounced narrowing from >~6 meV to ~2 meV (see Fig. 1F). The PL peak energies undergo two-step blueshift in the below- and above-threshold fluence ranges (see Fig. 1G). The below-threshold blueshift may be attributed to a reduction in the Rabi splitting energy caused by the screening effect from the increased reservoir exciton density, while the above-threshold blueshift likely results from repulsive or attractive interactions between polariton particles. The lower output of the second LP mode compared with the first LP mode arises from its higher dissipation channels, including partial relaxation into the first LP mode. No substantial differences in spectral narrowing or blueshift are observed between the first and second LP modes. These observations fulfill the essential criteria for polariton condensation at RT (*27*, *28*, *35*–*37*).

Figure S5 shows the results of full polarization tomography for the CsPbBr_3_ microcavity sample, which is identical to the sample discussed in Fig. 1. In the below-threshold pumping fluence range, as shown in fig. S5 (A and C), the LP modes are observed at ~2.308 and ~2.322 eV, those are horizontally (H) and vertically (V) polarized, respectively. This result arises from the orthorhombic crystal system of CsPbBr_3_ at RT (*28*, *33*, *34*). In the pump fluence range well above the threshold, as shown in fig. S5 (B and D), both the H- and V-polarized modes exhibit energy condensation. Notably, the condensed modes are not purely H or V polarized; instead, each mode includes both H- and V-polarized components. At the diagonal (D) and antidiagonal (A) polarization configurations [±45° linear polarizations, see fig. S5 (E to H)], as well as at the right (R) and left (L) circular polarization configurations [see fig. S5 (I to L)], PL signals from both the condensed modes are observed.

We reconfigure the results of polarization tomography in the form of Stokes parameters $S_{1}$, $S_{2}$, and $S_{3}$ defined as

$$S_{1}=\left(I_{H}-I_{V}\right)/\left(I_{H}+I_{V}\right)$$ (1)

$$S_{2}=\left(I_{D}-I_{A}\right)/\left(I_{D}+I_{A}\right)$$ (2)

$$S_{3}=\left(I_{R}-I_{L}\right)/\left(I_{R}+I_{L}\right)$$ (3)

**Fig. 2.**
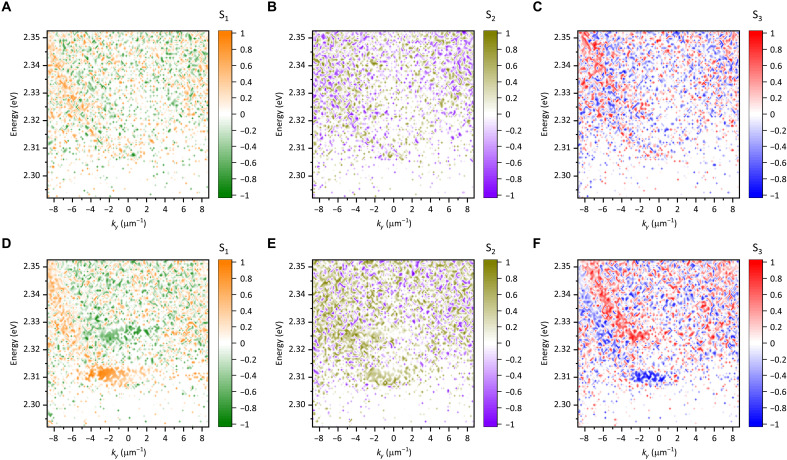
Colormaps of Stokes parameters obtained from full polarization tomography measurements for PL from CsPbBr_3_ microcavity. (**A** to **C**) Below-threshold ($P\sim 0.85P_{\text{th}}$) results for $S_{1}$ (A), $S_{2}$ (B), and $S_{3}$ (C) components. (**D** to **F**) Above-threshold ($P\sim 1.35P_{\text{th}}$) results for $S_{1}$ (D), $S_{2}$ (E), and $S_{3}$ (F) components.

Here, I is PL intensity at each polarization. As found in Fig. 2 (A to C), the below-threshold ($P\sim 0.85P_{\text{th}}$) polariton modes reveal almost only the $S_{1}$ components, being H or V polarized. As the emission intensity in the below-threshold regime is extremely weak, the signal-to-noise ratio of the polarization tomography is limited. As a result, the reconstructed Stokes maps in this regime may contain small pixel-level fluctuations originating from detector noise and sequential acquisition of the polarization-resolved images. On the other hand, the above-threshold ($P\sim 1.35P_{\text{th}}$) polariton modes contain not only the $S_{1}$ component but also large contributions from the $S_{2}$ and $S_{3}$ components (Fig. 2, D to F), undergoing the polarization superposition in the condensed LP modes and resulting elliptically polarized emission from them. The opposite parity observed for the $S_{1}$ and $S_{3}$ components, while the $S_{2}$ component shows the same parity between the two condensed modes, is similar to a spinor condensation behavior previously reported in an inorganic semiconductor microcavity (*19*). Figure S6 presents interferometric measurements of the elliptically polarized emission using a Michelson interferometer with a retroreflector configuration. The real-space interference image and the corresponding visibility profile indicate phase correlation between spatially inverted emission components, while the delay-dependent visibility yields a temporal coherence time of ~4 ps. These interferometric results provide strong experimental support for the identification of the observed emission as spinor condensate radiation. We note that, unlike earlier studies on CsPbBr_3_ microcavities that did not analyze the full Stokes parameters, including the $S_{2}$ component (*28*, *33*), our full-Stokes approach allows us to resolve the spinor nature of the condensed modes.

**Fig. 3.**
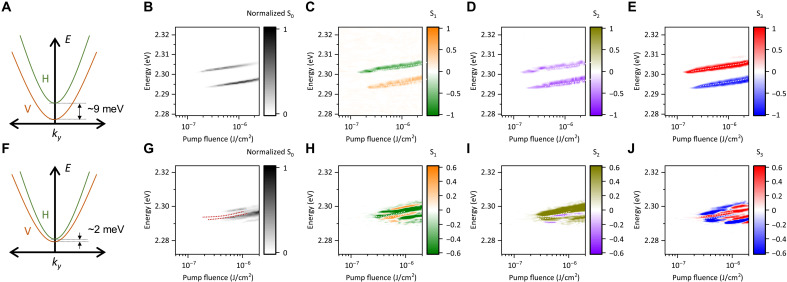
Pump fluence dependence of polariton condensation and spinor condensation. Results of CsPbBr_3_ microcavities with H-V splitting of ~9 meV (**A** to **E**) and ~2 meV (**F** to **J**). [(A) and (F)] Schematics of H-V splitting in LP mode. Fluence dependencies of $S_{0}$ [(B) and (G)], $S_{1}$ [(C) and (H)], $S_{2}$ [(D) and (I)], and $S_{3}$ [(E) and (J)].

Our recent study on CsPbBr_3_ polariton also confirmed the presence of polarization superposition in the condensed mode (*28*). While only the $S_{1}$ and $S_{2}$ components were discussed in that work, subsequent investigations revealed that a slight increase in pump fluence leads to the emergence of a nonzero $S_{3}$ component. This implies that the Stokes parameters evolve as a function of pump fluence, although there may be individual differences from sample to sample. To verify this hypothesis, we performed a more detailed analysis of the fluence-dependent polarization tomography for microcavity samples with various splitting energies. Figure 3 shows pump fluence–dependent polariton condensation emission for another CsPbBr_3_ microcavity. For this sample, the H-V splitting of LP mode is as large as ~9 meV (see Fig. 3A) where the variation in splitting energy among samples is more likely attributed to differences in the detuning between the cavity mode and the exciton energy (*34*), rather than to the crystal-fabrication method. Variation in the magnitude of splitting energy between samples is likely caused by the difference in detuning of the cavity mode from the exciton energy. Above ~0.2 μJ/cm^2^, as displayed in Fig. 3B, the unpolarized emission intensities $S_{0}$ ($=I_{H}+I_{V}$) exhibit substantial increase accompanied by the energy blueshifts with increased pump fluence. The observed threshold of ~0.2 μJ/cm^2^ is much smaller than the results shown in Fig. 1; this is because the samples fabricated by the solution-based method have DBRs with higher reflectivity than the samples fabricated by the vapor-phase method (see fig. S2). Figures 3C shows that the first and second LP modes have positive and negative $S_{1}$ components, respectively. The other components also show the trends consistent with those observed in Fig. 2; specifically, $S_{2}$ exhibits the same parity between the two modes, while $S_{3}$ reveals a pronounced spin splitting [see Fig. 3 (D and E)]. The original colormap data corresponding to Fig. 3 (C to E) are provided in figs. S7 to S9, respectively. We also evaluate another microcavity sample exhibiting a much smaller H-V splitting (~2 meV), as shown in Fig. 3F. For this sample, in addition to the main LP modes, several satellite modes appear at the high pump fluence region (see Fig. 3G). These satellite modes likely correspond to the higher-order polariton condensed mode with different angular momentum (*34*) or other polariton modes coupled to photonic modes with different orders. The existence of the satellite modes complicates the evolution of polarization states [see Fig. 3 (H and I)]. Nevertheless, the qualitative behavior of the Stokes parameters remains consistent with that observed in the other sample. In particular, the spin splitting between the two modes is evident (see Fig. 3J). These results demonstrate that the emergence of spinor condensation is robust and does not strongly depend on the magnitude of the LP splitting energy.

To elucidate evolutions of Stokes parameters with increasing the pumping fluence and to characterize spinor condensation in CsPbBr_3_ polaritons, we use a numerical analysis on the basis of spin-dependent Gross-Pitaevskii equation for condensed polariton modes, coupled with a rate equation describing reservoir population (see note S1) (*14*, *17*–*20*, *22*, *38*–*41*). Our model incorporates spin-asymmetric polariton-polariton interactions, with repulsive interaction (g > 0) for parallel spins and attractive interaction ($g_{12}$ < 0) for antiparallel spins. We consider a spatially uniform condensate under nonresonant continuous-wave (cw) excitation. As shown in fig. S6, the steady-state solution of the model reveals a double-threshold behavior as a function of the pumping fluence: The first threshold corresponds to normal polariton condensation, while the second marks the onset of spinor condensation. As the present experiments involve pulsed excitation and spatially nonuniform polariton densities, the simplified model is not intended to quantitatively reproduce the measured Stokes parameters but rather to illustrate the qualitative mechanism underlying the two-stage threshold behavior.

**Fig. 4.**
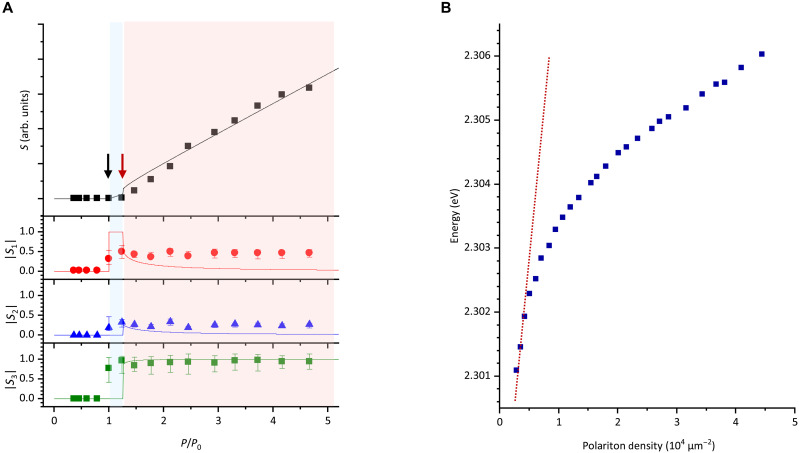
Characterization of RT spinor condensation. (**A**) Comparison of experimental results (symbols) and calculation results (solid curves). The calculation result was obtained by using the device parameters of Ω = 10 meV, $\delta g\left(=g-g_{12}\right)$ = 0.62 μeV·μm^2^, $\hbar \gamma_{c}$ = 23 meV (*Q* ~ 1000), $\hbar \gamma_{R}$ = 50 meV, and $\hbar \gamma_{d}$ = 6 meV. (**B**) Blueshift of condensed polariton mode. The horizontal axis is polariton density, which is estimated from the pump fluence under the assumption that all the excitation photon is absorbed for generating reservoir excitons. The error is within the size of the plotting symbols.

Figure 4A compares the experimental results, extracted from Fig. 3 (B to E), with the numerical calculation results obtained by substituting the appropriate device parameters (described later in detail) into eq. S19. The degree of polarization (DOP) evaluated from the experimental results is shown in fig. S10. We identify the explicit appearance of nonzero DOP as the first threshold. Meanwhile, the explicit increase in the S component is identified as the second thresholds. Although there are discrepancies in the amplitudes of the Stokes parameters $S_{1}$ to $S_{3}$ between the experiment and calculation, likely due to difference in the excitation regime (Gaussian-like density distribution under picosecond-pulsed excitation in the experiment versus spatially uniform density distribution under cw excitation in the calculation), we focus primarily on the qualitative reproduction of the threshold behavior rather than a quantitative agreement of the polarization amplitudes. We find that the experimental threshold behavior is reasonably well reproduced by the calculation, in which the second threshold $P_{\text{th}2}$ is ~1.2 times (or less) the first threshold $P_{\text{th}1}$. This result is similar to a previous study on a GaAs-based microcavity at cryogenic temperature (*19*).

Nevertheless, there are two critical differences between the GaAs-based microcavity and our system. The first one is the temperature at which spinor condensation is observed. The RT spinor condensation of CsPbBr_3_ microcavity is highly attractive for quantum applications. The second is the magnitude of H-V splitting of LP mode. In our system, the splitting energy Ω is as large as ~10 meV, whereas, in GaAs-based microcavity, it is as small as ~50 μeV (*19*). As described in note S2, the Gross-Pitaevskii analysis yields

$$\frac{P_{\text{th}2}}{P_{\text{th}1}}=1+\frac{2\Omega }{\delta g}\frac{R}{\gamma_{R}}$$ (4)

indicating that a larger Ω leads to a higher spinor condensation threshold. Here, R and $\gamma_{R}$ are the stimulated scattering coefficient and the damping constant, respectively, of the reservoir state. Despite the fact that Ω in the CsPbBr_3_ microcavity is two or three orders of magnitude larger than that in the GaAs-based system, the CsPbBr_3_ microcavity exhibits a comparable $P_{\text{th}2}$. This observation suggests that a substantially large $\left(g-g_{12}\right)$ plays a crucial role in enabling the low-threshold spinor condensation in CsPbBr_3_ microcavities.

We independently estimate the order of magnitude of the interaction coefficient $\left(g-g_{12}\right)$ of the CsPbBr_3_ microcavity using a conventional density-dependent blueshift analysis. As shown in Fig. 4B, the energy of condensed LP mode extracted from Fig. 3B exhibits a blueshift as a function of the two-dimensional polariton density. The slope of this relationship directly corresponds to $\left(g-g_{12}\right)$, representing the effective polariton-polariton interaction strength. Although a saturation trend due to screening effects becomes apparent at high densities, the low-density limit yields an estimated interaction strength on the order of $\left(g-g_{12}\right)$ ~0.62 ± 0.01 μeV·μm^2^ or higher (see red dashed line). This order-of-magnitude estimation suggests that the interaction strength in the CsPbBr_3_ microcavity is substantially larger than that typically reported for GaAs-based microcavities (~0.07 μeV·μm^2^) (*42*). The calculation result presented in Fig. 4A shows good agreement with the experimental threshold ratio $P_{\text{th}2}<\sim 1.2P_{\text{th}1}$, using $\left(g-g_{12}\right)$ = 0.62 μeV·μm^2^ and $\hbar \gamma_{R}$ = 50 meV, indicating that the interaction strength estimated from the experiment is consistent with the qualitative threshold behavior reproduced by the model. The value of $\hbar \gamma_{R}$, corresponding to the linewidth of excitonic absorption peak (see fig. S1), is ~20 times larger than that of GaAs quantum wells at cryogenic temperature (*43*). It is therefore demonstrated that the ratios $P_{\text{th}2}/P_{\text{th}1}$ in GaAs-based system and our system are reasonable to be equivalent, assuming a comparable R. By contrast, as exhibited in fig. S11B, using a smaller $\left(g-g_{12}\right)$ value leads to an increased $P_{\text{th}2}$, resulting in a notable deviation from the experimental observation. These results strongly support the significance of polariton-polariton interactions in determining the spinor condensation threshold.

## DISCUSSION

In this study, we present experimental evidence for RT spinor polariton condensation in a lead-halide perovskite microcavity under nonresonant optical excitation. Full polarization tomography revealed the formation of circularly polarized condensates, providing direct evidence of spinor condensation at RT. Consistent with qualitative analysis based on a spin-dependent Gross-Pitaevskii model, our findings highlight that the strong polariton-polariton interactions in CsPbBr_3_ facilitate spinor condensation at a low threshold, even in the presence of substantial polarization mode splitting. It is important to note that, unlike previously reported circularly polarized condensates arising from optical Rashba-Dresselhaus–type spin-orbit coupling (*44*, *45*), the spinor behavior observed here originates from the intrinsic excitonic spin degrees of freedom in the material, representing a distinct physical mechanism. This excitonic origin also implies robust spin coherence and controllability at ambient conditions, offering clear advantages for potential applications in RT spin-based polaritonic devices and quantum photonic technologies.

It remains unclear whether the repulsive interaction g or the attractive cross-spin interaction $g_{12}$ plays a more dominant role in enabling the low-threshold spinor condensation observed in our system. As a possible scenario, we suggest that a Bardeen-Cooper-Schrieffer–like condensation mechanism may be relevant for CsPbBr_3_ polariton (*27*, *46*, *47*). In this context, a physical environment that enhances spin-correlations, which will be reflected in a large magnitude of $g_{12}$, might play a crucial role in promoting spinor condensation with a reduced threshold.

The findings in this study not only deepen our understanding of spinor condensate dynamics in perovskite-based systems but also establish a robust platform for the optical manipulation of polariton spin states at RT. Moving forward, this work opens exciting prospects for the design of polaritonic devices with spin-selective functionalities, including ultrafast optical switches, spin-based logic gates, and coherent spin transport systems. Furthermore, the material flexibility and scalability of lead-halide perovskites offer unique opportunities to integrate polariton-based quantum elements into on-chip photonic circuits. We anticipate that the realization of RT spinor condensation in perovskite microcavities will accelerate the development of next-generation polaritonic quantum technologies and contribute largely to the broader field of spin-based optoelectronics.

## MATERIALS AND METHODS

### Microcavity fabrication

We used DBRs that were multilayers of SiO_2_ and TiO_2_ deposited on BK7 glass plates (area of 10 mm by 10 mm and thickness of 0.5 mm). The electron-beam evaporation was used for deposition of nine pairs of SiO_2_ and TiO_2_ layers. The top DBR mirror had a reflection band (>98%) of ~450 to 550 nm. The bottom mirror had a reflection band of ~500 to 600 nm.

Cesium bromide (CsBr) and lead(II) bromide (PbBr_2_) were purchased from Tokyo Chemical Industry and used without further process. Dimethyl sulfoxide (DMSO) was obtained from Nacalai Tesque. For the modified solution-based cast-capping crystal growth, CsBr and PbBr_2_ were dissolved in DMSO at a 1:1 molar ratio to achieve a concentration of 0.4 M. The solution was stirred at 300 rpm in a screw-cap tube using a magnetic stir bar for ~15 to 25 min at 60°C. The DBR mirrors were cleaned sequentially by ultrasonic treatment in ethanol and acetone for 5 min each, followed by ultraviolet-ozone cleaning for 15 min. Subsequently, 10 μl of the supersaturated precursor solution was cast onto the bottom DBR mirror. The sample was then capped with the top DBR mirror and secured using a ~15-mm double clip. Next, 9 to 10 μl of acetonitrile as poor solvent was injected into the gap between the DBR mirrors using a micropipette from three sides of the sample to promote further crystal growth. The sample was left in a vacuum atmosphere at RT for several days to promote crystal growth. After that, the remaining DMSO solvent on the top- and bottom-surfaces was removed by rinsing with ethanol. The resulting crystal thickness, typically ranging from 300 to 700 nm, was influenced by the clipping pressure and the volume of solution cast during the initial step.

High-quality CsPbBr_3_ single crystals were synthesized using the modified vapor-phase crystal growth: an atmospheric pressure proximity deposition (APPD) method. The APPD setup consisted of a bottom silicon substrate with a 300-nm SiO_2_ layer and a top cover glass placed in close proximity within a custom-designed apparatus. Before crystal growth, the substrates were sequentially cleaned using ultrasonic baths in acetone, isopropanol, ethanol, and deionized water, with each step performed for 5 min and repeated twice. The substrates were then dried under nitrogen flow. The precursor powders, CsBr and PbBr_2_, were placed on a heated stage within the deposition chamber. The deposition was performed under ambient atmospheric conditions. The proximity of the top and bottom substrates promoted efficient crystal nucleation and growth. The crystal growth proceeded over several hours at controlled temperatures optimized for CsPbBr_3_ formation. After that, the crystal flakes were transferred onto a bottom DBR substrate by using a thermal release tape. The top DBR was deposited onto the CsPbBr_3_ crystals by the radio frequency–magnetron sputtering method. The DBR was 9.5 pairs of HfO_2_/SiO_2_ multilayer having a reflection band (>98%) of ~490 to 540 nm. The PL quantum yield (PLQY) of the CsPbBr_3_ crystal was evaluated by using a PLQY spectrometer (C11347, Hamamatsu) to be ~8.9 and ~0.8%, before and after the sputtering. Given that the low excitation density of our equipment, ~0.1 to 1 mJ/cm^2^, PLQY after sputtering process is not particularly poor for a perovskite crystal (*48*); rather, the PLQY before sputtering is exceptionally high.

### Characterization

All optical measurements were performed in atmospheric condition of ~23°C and ~40% in related humidity. We used a picosecond laser (PT403, EKSPLA) for sample excitation. The wavelength and pulse width are 355 nm and 15 ps, respectively. The repetition frequency was set at 1 kHz. The PL signal was corrected by objectives with numerical aperture (NA) of ~0.5 or ~0.75 (UPLFLN 20× or 40×, Olympus) and introduced to a charge-coupled device spectrometer (Kymera 328i and Newton DU971P, Oxford ANDOR). The emission counts were recorded with time-integration of 1 s and accumulation of two to five times. We used a Fourier space imaging setup to obtain the angle-resolved PL spectra. Polarization measurements were performed using Glan-Taylor prisms and λ/4 wave plates (Thorlabs or Edmund). The angle limit is estimated from a relationship of $\theta_{\text{max}}={\text{sin}}^{-1}\left(\text{NA}\right)$.

## Supplementary Materials

### This PDF file includes:

Figs. S1 to S11 Notes S1 and S2
