# Supplementary material for: Room-temperature spinor condensate in halide perovskite microcavity
Source: Sci Adv. 2026 May 22;12(21):eaeb1521. doi: 10.1126/sciadv.aeb1521 (PMC13196750; doi:10.1126/sciadv.aeb1521)
Supplement: Supplementary file 1 — Figs. S1 to S11 Notes S1 and S2 [file sciadv.aeb1521_sm.pdf]

Science Advances
AAAS

Supplementary Materials for

Room-temperature spinor condensate in halide perovskite microcavity

Takaya Inukai *et al.*

Corresponding author: Kenichi Yamashita, yamasita@kit.ac.jp

Sci. Adv. **12**, eaeb1521 (2026)
DOI: 10.1126/sciadv.aeb1521

This PDF file includes:

Figs. S1 to S11
Notes S1 and S2

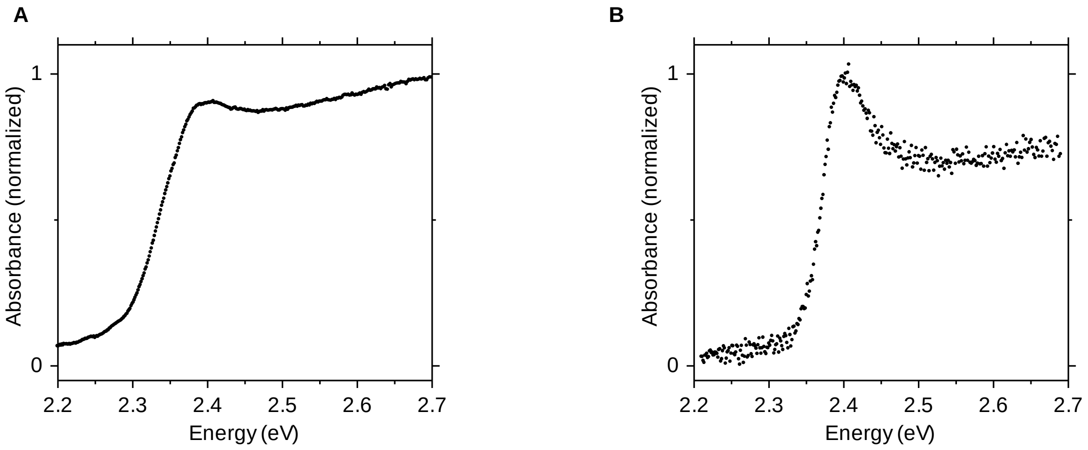

Figure S1. Absorption spectra of $CsPbBr_3$ crystals. (A) Spectrum of crystal grown by solution-based cast-capping-method.(*27*) (B) Spectrum of crystal grown by a modified vapor-phase chemical deposition method. These data were recorded by a home-made microscopic spectroscopy equipped with a halogen lamp as the light source. Both the spectra exhibit excitonic absorption peaks around ~2.4 eV, which corresponds to the exciton mode energy in an analysis of light-matter coupling.

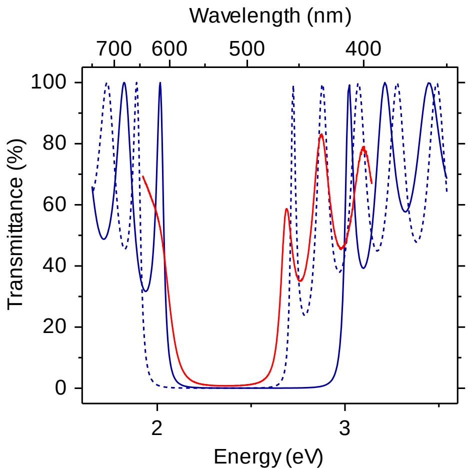

Figure S2. Transmittance spectra of high-reflectivity distributed Bragg reflectors (DBR) used for $CsPbBr_3$ microcavity. Solid blue and dashed blue curves show the data

for top- and bottom-DBRs, respectively, used in microcavities fabricated with the solution-based cast-capping method. the top- and bottom-DBRs. Red solid curve exhibits a transmittance spectrum of the top-DBR in a microcavity fabricated with the modifies vapor-phase chemical deposition method. The top-DBR was deposited by RF-magnetron sputtering method. The bottom-DBR of this microcavity is the same with that used in the microcavities fabricated with the solution-based cast-capping method.

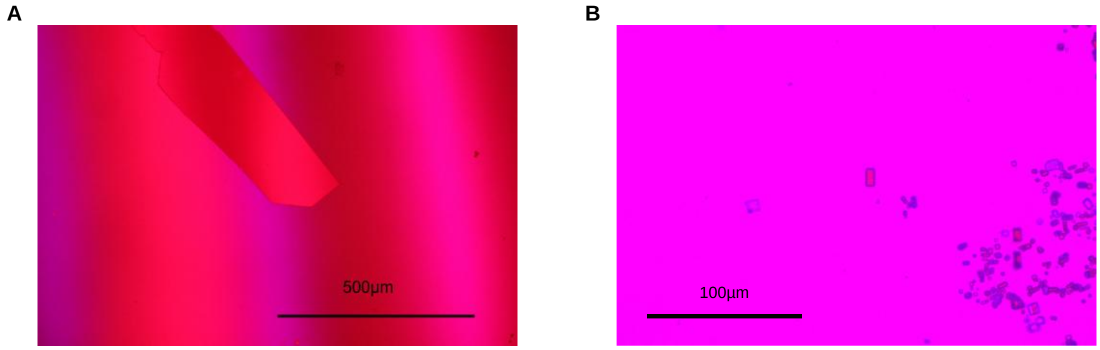

Figure S3. Microscopic images of $CsPbBr_3$ microcavities. Samples fabricated with cast-capping method (A) and modified vapor-phase chemical deposition method (B).

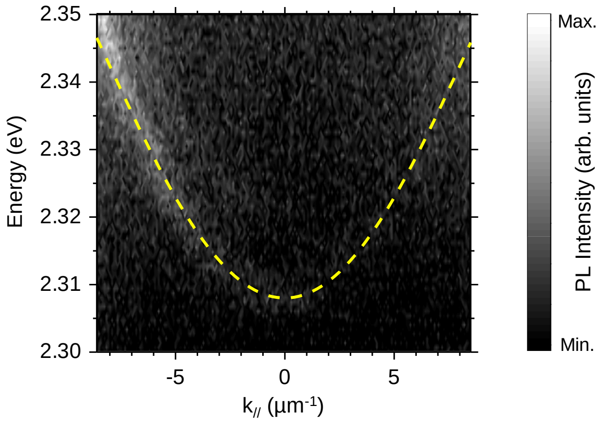

Figure S4. Polariton formation in $CsPbBr_3$ microcavity. A greyscale map of angular-

dependent photoluminescence result of $CsPbBr_3$ microcavity measured by a Fourier imaging spectroscopy. A yellow dashed curve shows a fitting function for lower polariton (LP) mode energy, leading to the Rabi-splitting energy of ~110 meV. The exciton energy was set to be ~2.4 eV in the analysis.

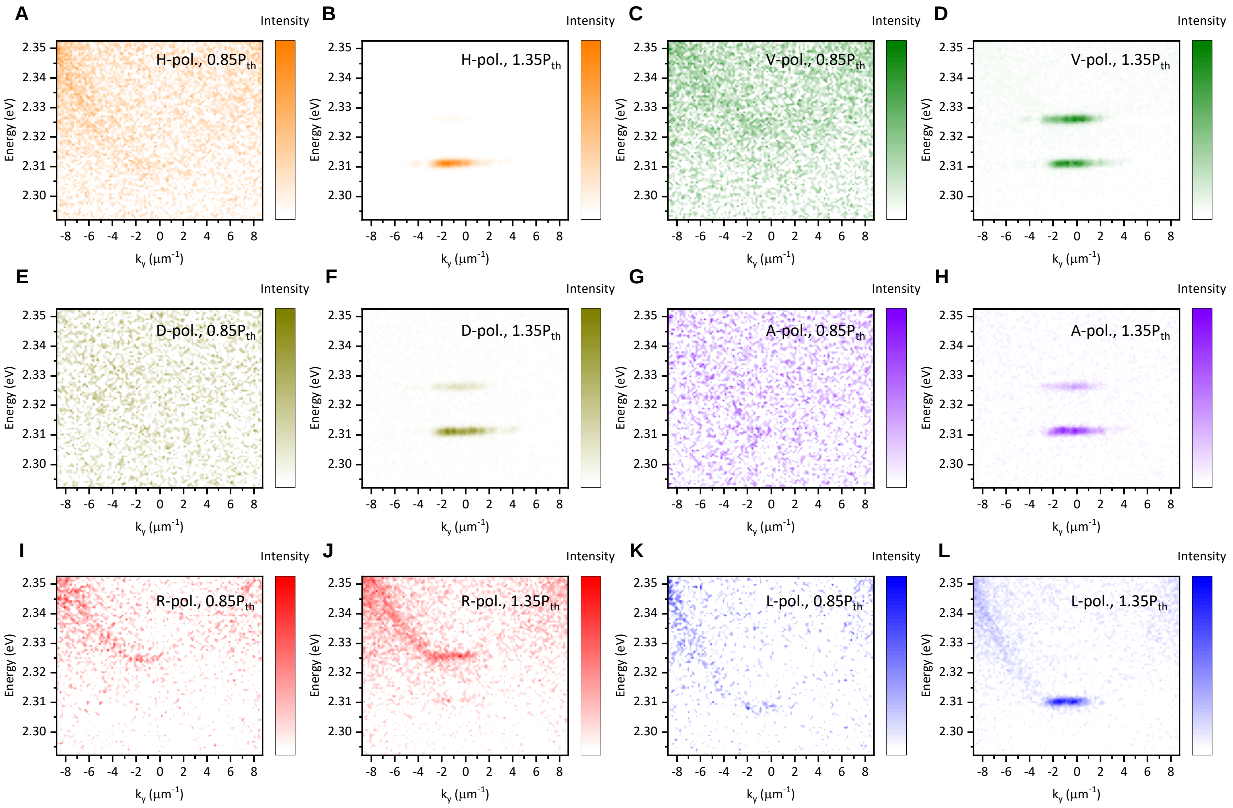

Figure S5. Polarization-dependent photoluminescence of a $CsPbBr_3$ microcavity. Results of horizontally (A, B), vertically (C, D), diagonally (E, F), anti-diagonally (G, H), right-circularly, (I, J), and left-circularly (K. L) polarized emission at below-threshold (A, C, E, G, I, K, ~$0.85P_{th}$) and above-threshold (B, D, F, H, J, L, ~$1.35P_{th}$) are shown (P_{th} ~ 14.3 μJ/cm^2). While panels (I) and (K) indicate precursors to spinor condensation, the polariton system remains in the below-threshold regime, leading to spin-isotropic

polarization, as shown in Fig. 2C of the main text.

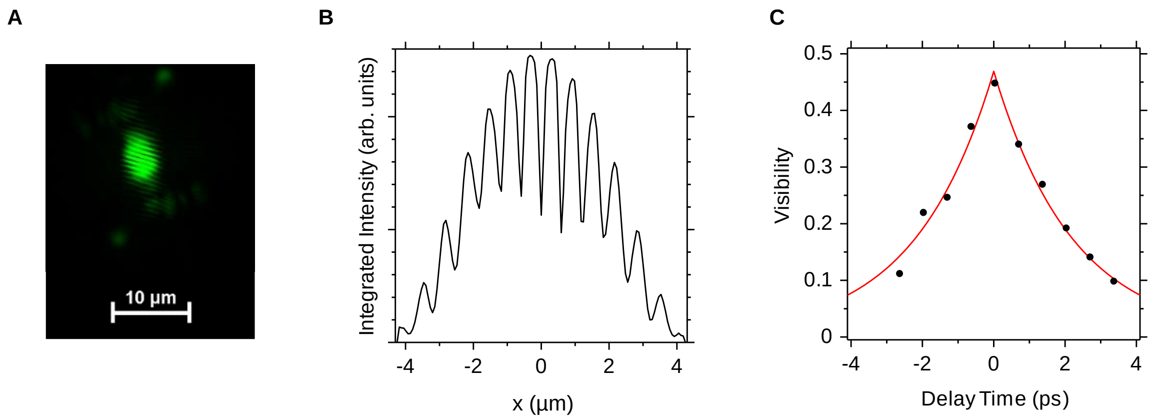

Figure S6. Interferometric characterization of the elliptically polarized emission. No wavelength filtering is applied in this measurement, but emission from the first LP mode is dominant. (A) Real-space interference image of the elliptically polarized emission obtained using a Michelson interferometer with a retroreflector in one arm, where emission from spatially inverted positions is superposed. (B) Spatial profile of the fringe visibility extracted from the interference image, demonstrating phase correlation between the inverted spatial components. (C) Delay-time dependence of the fringe visibility measured by introducing an optical delay in one interferometer arm, from which a temporal coherence time of ~4 ps is extracted.

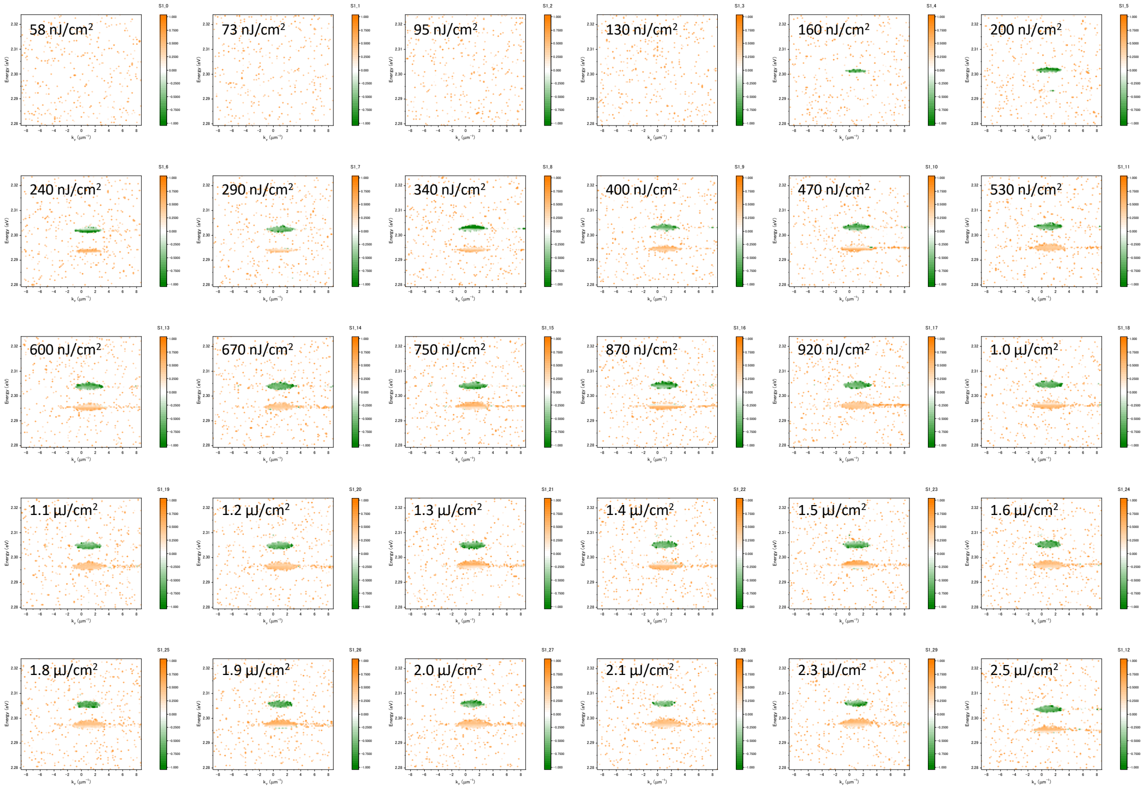

Figure S7. Full colormap data of fluence-dependent S_1. These are original data for Fig. 3C in the main manuscript. The pump intensity is lowest in the upper-left panel and increases progressively from left to right and from top to bottom.

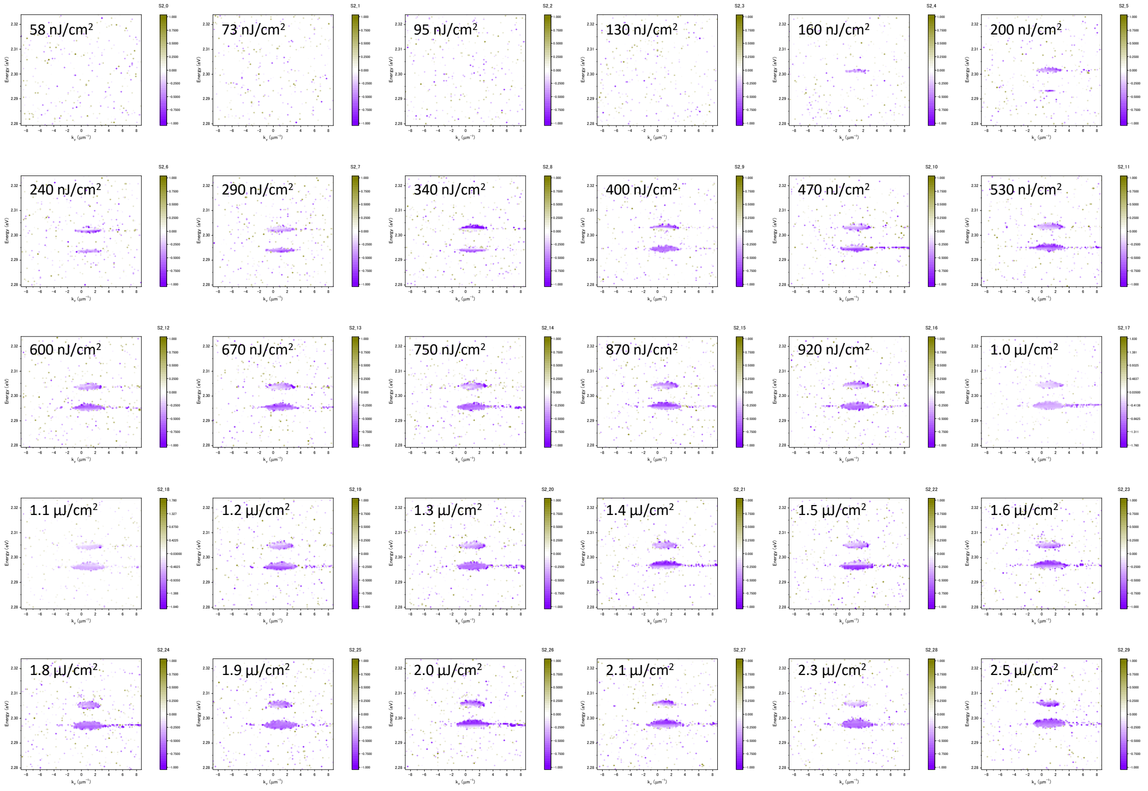

Figure S8. Full colormap data of fluence-dependent S_2. These are original data for Fig. 3D in the main manuscript. The pump intensity is lowest in the upper-left panel and increases progressively from left to right and from top to bottom.

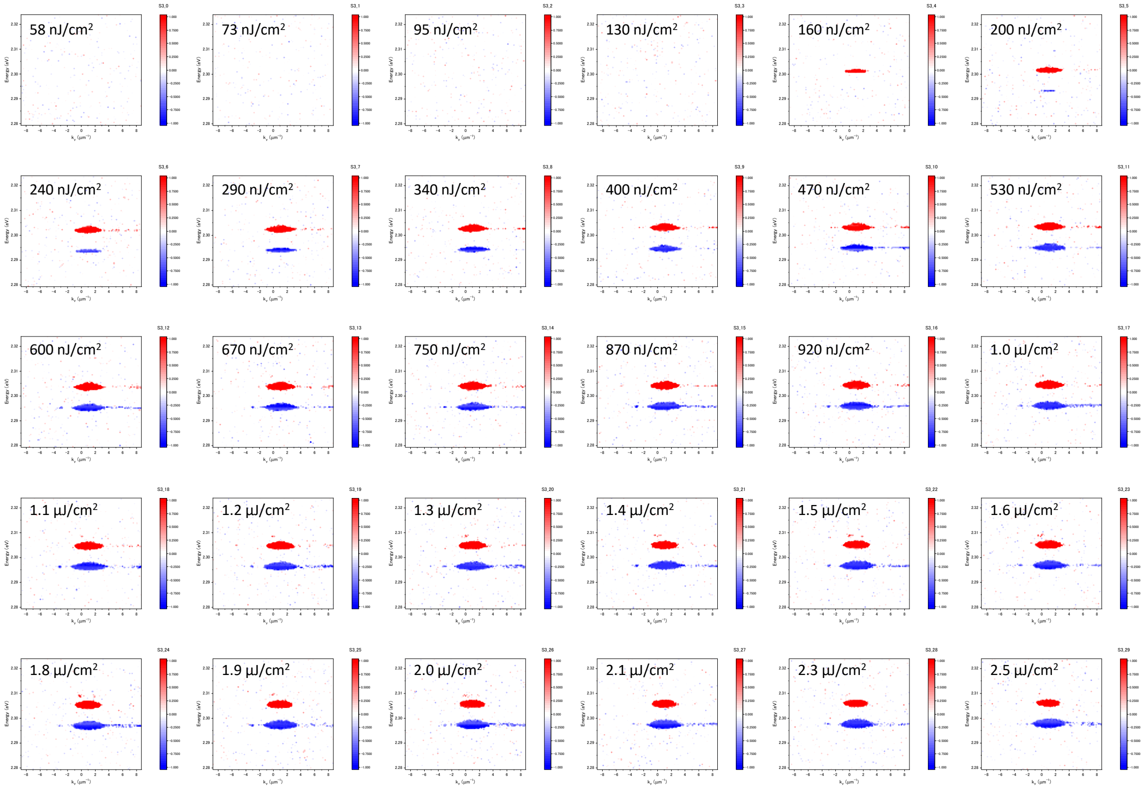

Figure S9. Full colormap data of fluence-dependent S_3. These are original data for Fig. 3E in the main manuscript. The pump intensity is lowest in the upper-left panel and increases progressively from left to right and from top to bottom.

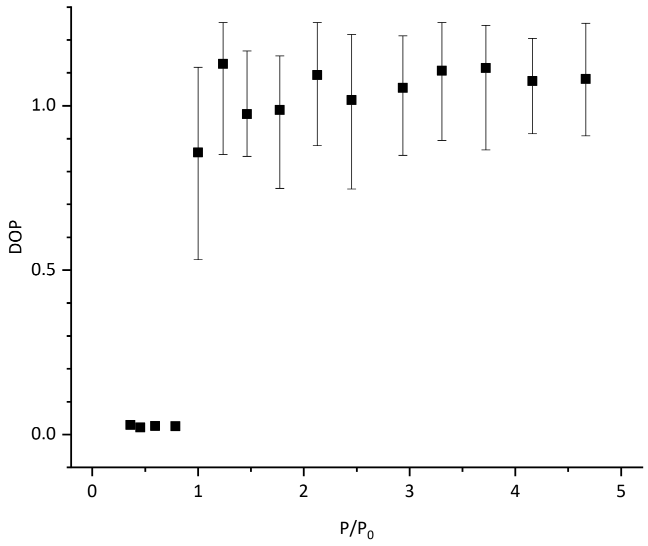

Figure S10. Degree of polarization (DOP) for the spinor condensation emission. The data are reconstructed from Fig. 4A of the main manuscript. Below the threshold ($P/P_0 < 1$), DOP lies in almost zero, although the above-threshold P/P_0 leads to DOP to approximately 1. The error estimated from averaging process of Stokes parameters is attributable to fluctuations in the excitation pump fluence, which cause variations in the original emission intensity with each polarization state.

Supplementary Note S1

Gross-Pitaevskii formalism of spinor condensation

Spinor condensation of a polariton mode in a microcavity with anisotropic cavity material is described by spin-dependent Gross-Pitaevskii equations (*14,20,25,27,39,41*). When the degeneracy of orthogonally polarized lower polariton (LP) modes are lifted due to the birefringence of the cavity material and the dissipation rates of split LP modes are not

equal, the spin-dependent Gross-Pitaevskii equations are written as (*25, 41*)

$$i\hbar\frac{\partial\psi_1}{\partial t}=\left[-\frac{\hbar^2\nabla^2}{2m}+g|\psi_1|^2+g_{12}|\psi_2|^2+g_R n_R\right]\psi_1+(\Omega-i\hbar\gamma_d)\psi_2$$

$$+\frac{i\hbar}{2}(Rn_R-\gamma_C)\psi_1 \quad \text{(S1)}$$

$$i\hbar\frac{\partial\psi_2}{\partial t}=\left[-\frac{\hbar^2\nabla^2}{2m}+g|\psi_2|^2+g_{12}|\psi_1|^2+g_R n_R\right]\psi_2+(\Omega-i\hbar\gamma_d)\psi_1$$

$$+\frac{i\hbar}{2}(Rn_R-\gamma_C)\psi_2. \quad \text{(S2)}$$

Here, ψ_1 and ψ_2 are the wavefunctions of polariton condensed modes with two different spin (circular) polarizations. m is the effective mass of polariton. g and g_{12} are the repulsive and attractive interaction constants between polariton particles with the same and the opposite spins, respectively, and thus we state as $g>0$ and $g_{12}<0$. γ_C is the decay rate of condensed polariton. Ω (>0) shows the coherent spin-flipping, which arises from the anisotropy-induced splitting of the LP modes. The term $-i\hbar\gamma_d$ exhibits the dissipative coupling caused by the difference between the dissipation rates of the split LP modes with orthogonal linear polarizations. The Gross-Pitaevskii equations (S1) and (S2) are also coupled to the rate equation of reservoir state with the density of n_R, i.e.,

$$\frac{\partial n_R}{\partial t}=P-\gamma_R n_R-R(|\psi_1|^2+|\psi_2|^2)n_R. \quad \text{(S3)}$$

Here, P is non-resonant and continuous pumping rate. γ_R and R are the dissipation rate of reservoir state and the stimulated scattering rate of reservoir state into the condensed states, respectively. Note that we consider the spatially uniform polariton condensation regime.

We introduce the pseudospin representation by using ψ_1 and ψ_2 as

$$S_x = \frac{1}{2}(\psi_1\psi_2^* + \psi_1^*\psi_2), \quad \text{(S4)}$$

$$S_y = \frac{i}{2}(\psi_1\psi_2^* - \psi_1^*\psi_2), \quad \text{(S5)}$$

$$S_z = \frac{1}{2}(\psi_1\psi_1^* - \psi_2^*\psi_2), \quad \text{(S6)}$$

$$S = \frac{1}{2}(\psi_1\psi_1^* + \psi_2^*\psi_2). \quad \text{(S7)}$$

It is easy to see that S is proportional to the density of polariton condensed state and corresponds to $\sqrt{S_x^2 + S_y^2 + S_z^2}$. Furthermore, S_x/S, S_y/S, and S_z/S correspond to Stokes parameters S_1, S_2, and S_3, respectively, which have been obtained experimentally from the full polarization tomography measurements. We consider the time derivatives of eqs. (S4) – (S7) by utilizing eqs. (S1) and (S2). As a result, the following set of rate equations can be obtained.

$$\dot{S_x} = -(\gamma_c - Rn_R)S_x - \frac{2}{\hbar}\delta g S_z S_y - 2\gamma_d S \quad \text{(S8)}$$

$$\dot{S_y} = -(\gamma_c - Rn_R)S_y - \frac{2}{\hbar}\Omega S_z + \frac{2}{\hbar}\delta g S_z S_x \quad \text{(S9)}$$

$$\dot{S_z} = -(\gamma_C - Rn_R)S_z + \frac{2}{\hbar}\Omega S_y \quad \text{(S10)}$$

$$\dot{S} = -(\gamma_C - Rn_R)S - 2\gamma_d S_x \quad \text{(S11)}$$

$$\dot{n_R} = P - (\gamma_R + 2RS)n_R \quad \text{(S12)}$$

Here $\delta g = g - g_{12}$. These rate equations give different steady-state solutions in the three regimes for the increased P, representing two types of threshold-like behavior. In the following analysis, solutions that are mathematically derivable but not physically significant are eliminated.

Let us start the discussion from the below-threshold incoherent regime where $S = 0$ $(S_x = S_y = S_z = 0)$. From eq. (S12), we find n_R is linearly increased with P. The set of steady-state solutions in this regime are as follows

$$\begin{cases} n_R = \dfrac{P}{\gamma_R} \\ S = 0 \\ S_x = 0 \\ S_y = 0 \\ S_z = 0 \end{cases}. \quad \text{(S13)}$$

Above the first threshold P_{th1} shown as

$$P_{th1} = \frac{\gamma_R}{R}(\gamma_C + 2\gamma_d), \qquad \text{(S14)}$$

n_R becomes constant and S shows a linear increase with P, as analogous to the conventional polariton lasing characteristics. However, S_y and S_z remain zero, revealing that the polariton condensed state is linearly polarized but not spin polarized. Eqs. (S8), (S11), and (S12) are available in this regime, leading to the following set of steady-state solutions.

$$\begin{cases} n_R = \dfrac{\gamma_C + 2\gamma_d}{R} \\ S = \dfrac{P}{2(\gamma_C + 2\gamma_d)} - \dfrac{\gamma_R}{2R} \\ S_x = \dfrac{P}{2(\gamma_C + 2\gamma_d)} - \dfrac{\gamma_R}{2R} \\ S_y = 0 \\ S_z = 0 \end{cases} \quad \text{(S15)}$$

Further increasing P, the polariton state moves to the third regime. All of the components shown in eqs. (S8) – (S12) show non-zero values, exhibiting the emergence of spin-polarized polariton condensation. We obtain the following relationships from eqs. (S11) and (S12).

$$S_x = -\frac{(\gamma_C - Rn_R)}{4R\gamma_d}\left(\frac{P}{n_R} - \gamma_R\right) \quad \text{(S16)}$$

From eq. (S9), on the other hand, we find

$$(\gamma_c - Rn_R)S_y = \frac{2}{\hbar}(-\Omega + \delta g S_x)S_z. \quad \text{(S17)}$$

Under the assumption that all the pseudospin components are not zero, we obtain

$$-\Omega + \delta g S_x = 0. \quad \text{(S18)}$$

Note that $(\gamma_c - Rn_R) = 0$ leads to $S = 0$ and thus is not correct in this regime. By substituting eq. (S16) into eq. (S18), we can derive the non-divergent steady-state solution for n_R as

$$n_R = \frac{\delta g(PR + \gamma_R\gamma_C) - 4\Omega R\gamma_d}{2\delta g R\gamma_R} - \frac{\sqrt{[\delta g(PR + \gamma_R\gamma_C) - 4\Omega R\gamma_d]^2 - 4PR(\delta g)^2\gamma_R\gamma_C}}{2\delta g R\gamma_R}. \quad \text{(S19)}$$

As a result, we obtain the following set of steady-state solutions for this regime.

$$\begin{cases} n_R = \dfrac{\delta g(PR + \gamma_R\gamma_C) - 4\Omega R\gamma_d}{2\delta g R\gamma_R} - \dfrac{\sqrt{[\delta g(PR + \gamma_R\gamma_C) - 4\Omega R\gamma_d]^2 - 4PR(\delta g)^2\gamma_R\gamma_C}}{2\delta g R\gamma_R} \\ S = \dfrac{1}{2R}\left(\dfrac{P}{n_R} - \gamma_R\right) \\ S_x = -\dfrac{(\gamma_C - Rn_R)}{2\gamma_d}S \\ S_y = \pm\dfrac{\hbar}{2}\sqrt{\dfrac{(Rn_R - \gamma_C)[(\gamma_c - Rn_R)S_x + 2\gamma_d S]}{\delta g\Omega}} \\ S_z = \pm\sqrt{\dfrac{\Omega[(\gamma_c - Rn_R)S_x + 2\gamma_d S]}{\delta g(Rn_R - \gamma_C)}} \end{cases} \quad \text{(S20)}$$

By substituting eq. (S20) into eq. (S15), we obtain the second threshold P_{th2} as

$$P_{th2} = \left(\frac{\gamma_R}{R} + \frac{2\Omega}{\delta g}\right)(\gamma_C + 2\gamma_d). \quad \text{(S21)}$$

Supplementary Note S2

Characterization of spinor condensate utilizing numerical calculation

In Fig. S11, we show some examples of numerical calculation result using eq. (S19). The P dependencies of n_R, S, and Stokes parameters $S_1 - S_3$ exhibit double-threshold-like behaviors. At the first threshold ($P \sim P_{th1}$), which corresponds to the conventional polariton condensation threshold, the monotonic increase of reservoir exciton density is inherited to that of polariton condensation phase. The polariton condensation mode is fully polarized with the horizontal (or vertical) polarization component ($S_1 = 1$). At the second threshold ($P \sim P_{th2}$), stimulation from the reservoir state to the polariton state becomes more significant, leading to modifications of P-dependent behaviors for n_R and S. Simultaneously, the S_2 and S_3 components appear, indicating the spin-polarized polariton condensed state and elliptically polarized emission. The non-zero S_2 component demonstrates the occurrence of the second symmetry breaking, locking their polarization state to a different state containing an S_2 component. The further increase of P causes a gradual transition to pure circularly polarized polariton mode.

From eqs. (S14) and (S21) describing P_{th1} and P_{th2}, respectively, we obtain

$$\frac{P_{th2}}{P_{th1}} = 1 + \frac{2\Omega}{\delta g}\frac{R}{\gamma_R}. \qquad \text{(S22)}$$

This relationship reveals that spinor condensation threshold is largely affected by two parameters, when the first threshold is constant. For example, as shown in Figs. S11A and S11B, the increased $\delta g\ (= g - g_{12})$ causes the decrease in the second threshold pumping fluence. The proximity of the first and second thresholds means that spin polarization is observable immediately after polariton condensation. The second threshold depends also on Ω; as shown in Figs. S11C and S11D, the larger Ω caused the higher second threshold.

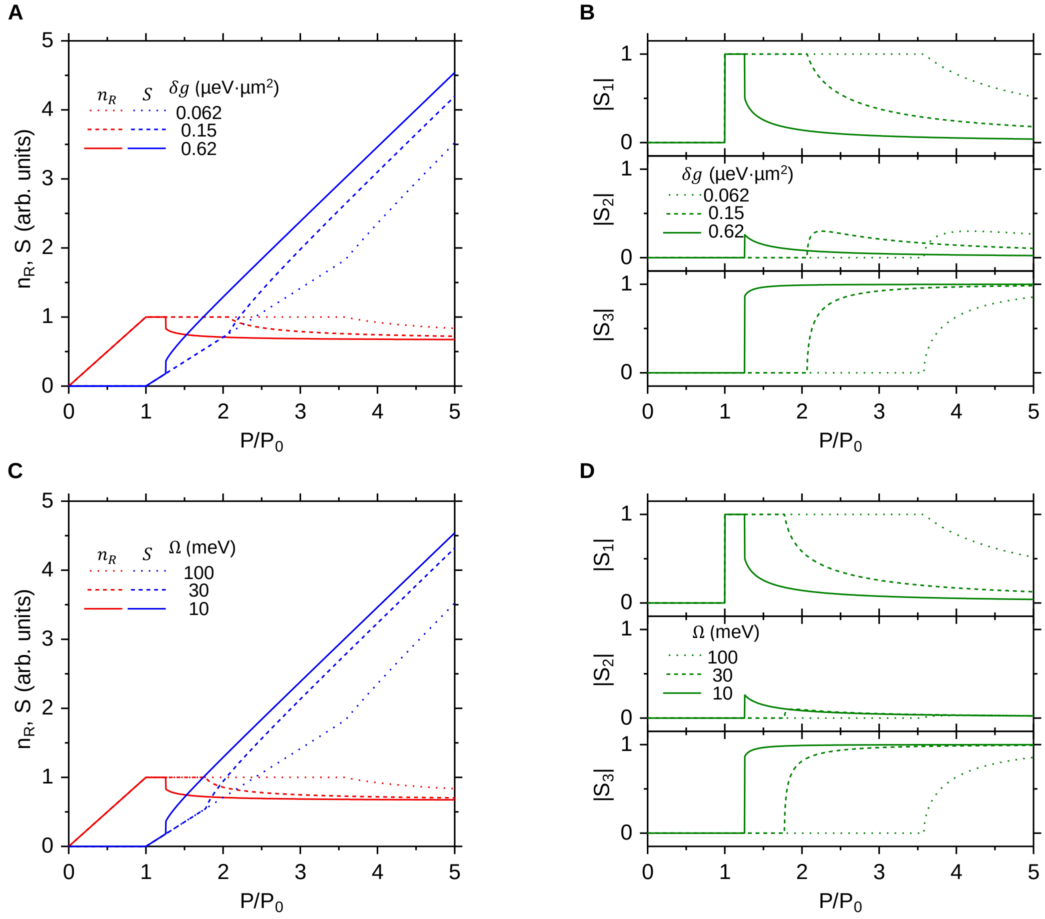

Figure S11. Pump fluence-dependent spinor polariton condensation. The data are calculated with eqs. (S13), (S15), and (S20) at $\hbar\gamma_d$ = 6 meV, $\hbar\gamma_c$ = 23 meV, and $\hbar\gamma_R$ = 50 meV. (A, B) P-dependent n_R and S (A) and $|S_{1-3}|$ (B) at Ω = 10 meV. δg is varied in a range of 0.062 – 0.62 μeV·μm^2. (C, D) P-dependent n_R and S (C) and $|S_{1-3}|$ (D) at δg = 0.62 μeV·μm^2. Ω is varied in a range of 10 – 100 meV. P_0 is the pump fluence at the first threshold for a result calculated at Ω = 10 meV and δg = 0.62 μeV·μm^2. $\hbar\gamma_c$ and $\hbar\gamma_R$ were estimated from experimentally obtained parameters of microcavity Q factor and excitonic absorption linewidth, respectively.
